# A link prediction method for MANETs based on fast spatio-temporal feature extraction and LSGANs

**DOI:** 10.1038/s41598-022-20981-3

**Published:** 2022-10-07

**Authors:** Hao Shao, Lunwen Wang, Hui Liu, Rangang Zhu

**Affiliations:** College of Electronic Engineering, National University of Defense Technology, Hefei, China

**Keywords:** Information theory and computation, Computer science, Mathematics and computing, Information technology

## Abstract

Link prediction aims to learn meaningful features from networks to predict the possibility of topology. Most of the existing research on temporal link prediction is mainly aimed at networks with slow topology changes. They ignore the information of topology interval and link duration. This paper proposes a link prediction model named FastSTLSG. It can automatically analyze the features of the topology in a unified framework to effectively capture the spatio-temporal correlation of Mobile Ad Hoc Networks. First, we regard the changing topology as a chaotic system, transform it into a series of static snapshots based on the autocorrelation function; Next, the fast graph convolutional network efficiently analyses the topological relationships between nodes and reduces the computational complexity by importance sampling. Then, the gate recurrent unit captures the temporal correlation between snapshots. Finally, the fully connected layer reconstructs the topological structure. In addition, we take full advantage of least squares generative adversarial networks to further improve the performance of generator to obtain high-quality link prediction results. Extensive experiments on different datasets show that our FastSTLSG model obtains higher prediction accuracy compared with existing baseline models.

## Introduction

Mobile Ad Hoc Networks (MANETs), as centerless, self-organizing, multi-hop wireless networks, consist of a set of mobile terminals carrying wireless transceiver devices^1^. Different from conventional networks, people can quickly establish the required mobile communication networks at any moment and place in the absence of existing network communication hardware. MANETs have been widely used in many fields, involving rescue and disaster relief, wireless medical monitoring systems, mobile office meetings and other fields^2–4^.

The movement of devices in MANETs leads to the generation or disappearance of links between nodes. The link relationships between devices change over time, causing the topology of the networks to evolve in continuous time steps^5^. Link prediction in MANETs aims to use the historical time-series topology to predict the future network structure. It allows us to learn appropriate MANETs structural evolution mechanisms, not only to gain insight into the connections between network topologies and functions, but also to analyze and control networks more precisely^6,7^.

Currently, existing link prediction methods focus on static networks. The similarity indices of nodes consider that the probability of link existence is positively correlated with the similarity of nodes. Common Neighbors (CN), Jaccard (JC), Salton, Admic Adar (AA), and Resource Allocation (RA)^8,9^ are all typical indices by comparing similarity of nodes in static networks. Further, Katz, Local Path (LP)^10^, and LHZ-II^11^ are based on path similarity, i.e., multi-order domain similarity of nodes, as prediction indices. The above methods have low computational complexity but cannot analysis the complex nonlinear features of the topological structure. To solve this problem, network embedding methods such as Node2vec^12^ and GraphWave^13^ have been proposed, which aim to convert the nodes into low-dimensional representations and apply the feature representations to various graph tasks. However, these link prediction models which ignore temporal information are still not suitable for prediction of time-series networks^14,15^. In recent years, several researchers have proposed models to predict future links based on historical topological data. Li^16^ proposes SLIDE which aims to maintain and update a low-rank sketch matrix to summarize historical data and use the sketch matrix to dynamically infer missing links. Moreover, with the development of deep learning, some Encoder to Decoder frameworks have been applied to temporal prediction of dynamic networks, such as E-LSTM-D^6^, DDNE^17^, FastGCRNN^18^ and TGNs^19^.

Based on GNN and RNN, researchers have proposed novel temporal link prediction models named DGFT^20^ and GGAN^21^, which have achieved excellent performance. However, these models still have the following shortcomings.

1. Most of the existing research on temporal link prediction is mainly aimed at networks with slow topology changes, such as social networks. MANETs, as networks with rapid topology changes. The ordinary models ignore the deep potential change factors of MANETs, while lacking the ability to capture both nonlinear spatial and temporal features efficiently. In addition, the above models do not study the characteristics of MANETs. The training process is inefficient and takes a long time.
2. Lack of the appropriate interval between each snapshot. Considering the rapid change of MANETs, determining a reasonable time interval for static snapshots is crucial to the accuracy of prediction. The above models simply take the changed topology as the input of deep learning frameworks. In Table 1, the topology of a dynamic network is named *A* in 1 s and 2 s. The network changes in 3 s, 4 s, 5 s, 6 s, 8 s, 9 s, and the topology is named *B*, *C*, *D*, *E*, *F*, *H* respectively. The inputs of the ordinary models are *A*, *B*, *C*, *D*, *E*, *F*, *H*. This will lead to the following negative effects: (i) The difference between each snapshot is very small because only one link has changed. When analyzing the rapidly changing large-scale MANETs, it will bring a large amount of redundant data, which is not conducive to the training of the model. (ii) The interval time of each snapshot is different. In existing models, each snapshot is simply regarded as equally interval data, ignoring the different link duration between them.

**Table 1:** The input of different temporal prediction models.

Time	1 s	2 s	3 s	4 s	5 s	6 s	7 s	8 s	9 s
Topology	*A*	*A*	*B*	*C*	*D*	*E*	*E*	*F*	*H*
**Input**									
Existing model	*A*		*B*	*C*	*D*	*E*		*F*	*H*
Our model	*G*_1_			*G*_2_			*G*_3_
3. Lack of prediction about link duration. The existing methods only predict the existence or non-existence of links at the future moment. In practical, the link duration in MANETs contains important information about the node behaviors and network state at the future time. The existing models regard continuous snapshots as equal interval data. Although we can predict the network as a certain topology, we cannot predict the duration of this topology, which limits the application of the models.
4. Most deep learning-based models utilize network embedding to mine features and capture the spatio-temporal dependencies. However, because of the sparsity of the network topology, it is difficult to accurately recover the original topology from low dimensional dense representation data^17^. Based on the embedding data, how to enhance the ability of the model to reconstruct network snapshots is one of the problems we need to address.

To solve the above problems, we propose a link prediction model for MANETs called FastSTLSG, which is based on fast spatio-temporal feature extraction and LSGANs. We regard MANETs as a kind of chaotic system, refer to the phase space reconstruction technology^22^ of coordinate delay in chaotic time series theory, use autocorrelation function to determine an appropriate interval of snapshots, and take the device connection duration as the link weight. In Table 1, we slice the MANETs into multiple fixed interval snapshots (*G*_1_, *G*_2_, *G*_3_, *G*_4_, *G*_5_), and take these five snapshots as the inputs of the proposed model. On this basis, Fast Graph Convolutional Networks (FastGCN)^23^ and stacked Gated Recurrent Unit (GRU)^24^ are used to efficiently process the high-dimensional and nonlinear historical structure data of MANETs. To improve the performance of generator, we construct a generator and a discriminator based on Least Square Generative Adversarial Networks (LSGANs)^25^, obtain high-quality generator and accurate prediction results of MANETs by adversarial training. In addition, we also construct penalty terms to guide the model to generate existing links rather than nonexistent ones in the corresponding position of the adjacency matrix, to avoid the negative impact of topology sparsity. In short, the main contributions of this paper are as follows.

1. Adaptive slicing time calculation. We are the first to use chaotic time series theory to determine the slicing time of MANETs and use the link duration within different snapshots as link weights. Our model has the following advantages. (i) It reduces redundant data and compresses input data. More than one link may change between adjacent snapshots. The reduction of redundant data will prevent the model from extracting useless information. The data compression can make the model better adapt to the rapid changes of topology. (ii) The time interval between adjacent snapshots is equal, which avoids the negative impact of different link connection duration on graph feature extraction. (iii) Based on the slicing time, we can predict the connection duration of each link in the next time interval.
2. High-quality generator. FastGCN and GRU are used to capture the consistency of spatio-temporal features, while considering the network structure and evolution pattern of each time. In addition, we improve the prediction performance of the model by exploiting the adversarial training based on LSGANs. Besides adversarial training, we also use reconstructing loss and penalty matrix to balance the negative effect of sparsity, and finally generate high-quality prediction results based on historical structure data of MANETs.
3. Better performance than previous works. We evaluate the proposed model FastSTLSG on real MANETs datasets and compare it with several existing baseline methods. The results show that our model outperforms all competitors.

## Related works

### Link prediction for MANETs

**Figure 1:**
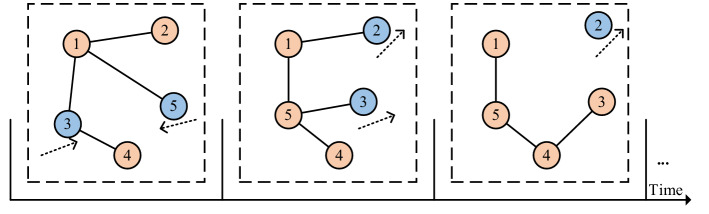
A brief model of MANETs.

Figure 1 represents a brief model of MANETs in which different nodes move in the direction of arrows at different times, resulting in changes in the topological structure^1–4^.

We define MANETs as a series of network snapshots $G = \{ G1,G2 \cdots ,Gs\} ,i \in \{ 1,2, \ldots ,s\}$, where *i* represents the serial number of the snapshots and the *i*-th network snapshot $Gi$ is represented by $Gi{ = (}V,Ei,Wi{)}$. The all snapshots have the same node set *V*, $Ei$ and $Wi$ are link set and weight set, respectively. Considering that the adjacency matrix **A** can completely describe the topological structure of the snapshot, a series of ${\mathbf{A}}i$ are used as input and output data of the prediction model FastsSTLSG.

In static networks, link prediction aims to analyze the links that exist but have not yet been discovered based on the observed topology^26^. Approximately, the link prediction in MANETs uses the information extracted from the previous network topology to reveal evolution pattern of networks^27^. In short, the purpose of link prediction for MANETs is to predict the links that appear or disappear in the next time stage, that is, the network snapshot $Gs + 1$, based on the previously observed networks $\{ G1,G2, \cdots ,Gs\}$ of length *s*.

### GCN

Convolutional Neural Network (CCN) has strong feature extraction and integration capabilities when processing image data, thanks to the parameter sharing and weighted averaging of convolution kernels^28^. However, the network topology belongs to non-Euclidean data, that is, the number of neighbor nodes of each node in graph is not necessarily the same. To solve this problem, researchers have exquisitely designed a variant of CNN to extract features from non-Euclidean structured data, named GCN, which can operate directly on graphs^29,30^.

Suppose there is a static network $G(V,E)$ composed of $\left| V \right|$ nodes, and the $\left| V \right| \times \left| V \right|$ dimensional adjacency matrix **A** is composed of the link relationships between $\left| V \right|$ nodes. If each node has $\left| M \right|$ dimensional features, the feature matrix **Z** is $\left| V \right| \times \left| M \right|$ dimensional. The **A** and **Z** are the input data of the GCN, the convolution process between layers as follows:

1 $$ {\mathbf{H}}^{l + 1} = \sigma ({\tilde{\mathbf{D}}}^{ - 1/2} {\mathbf{\tilde{A}\tilde{D}}}^{ - 1/2} {\mathbf{H}}^{(l)} {\mathbf{W}}^{(l)} ) $$

where ${\tilde{\mathbf{A}}} = {\mathbf{A}} + {\mathbf{I}}$, ${\mathbf{I}}$ is unit matrix, degree matrix ${\tilde{\mathbf{D}}} = \sum\nolimits_{u} {{\tilde{\mathbf{A}}}uv}$, ${\mathbf{H}}$ is the feature matrix in each layer, ${\mathbf{W}}^{(l)}$ represents the weight matrix to be trained in the *l*-th layer, $\sigma$ is a nonlinear activation function.

GCN has achieved good performance in many graph tasks, but it has poor scalability because GCN is a transductive learning method. In MANETs, the generation of new nodes and the change of links make it difficult to extend GCN to networks with unknown topological structures. When using GCN to train some networks with high density, neighbor extension of an exceedingly small number of nodes will contain a large portion of the full graph in a noticeably short time, which can bring a huge computation cost. In fact, the ordinary GCN cannot satisfy the application in the fast-changing MANETs.

## Methodology

**Figure 2:**
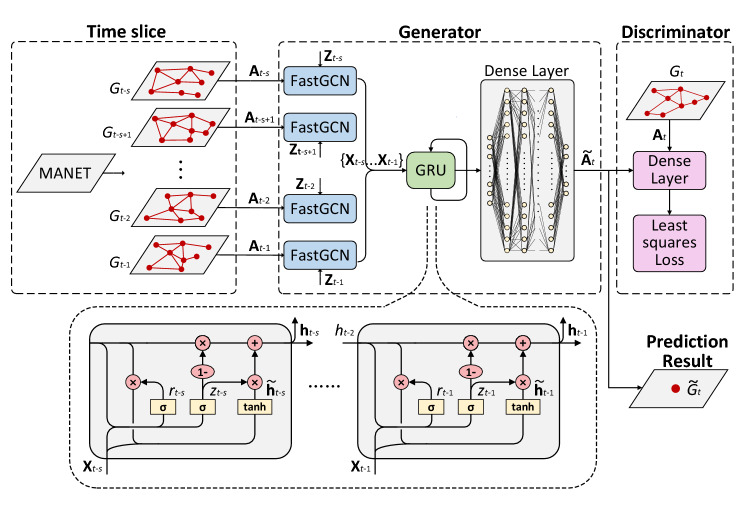
The framework of FastSTLSG.

In this section, we introduce in detail the link prediction model for MANETs named FastSLSG proposed in this paper. The framework of FastSTLSG is shown in Fig. 2, which is mainly divided into three units: (1) Time Slice; (2) Generator (including FastGCN, GRU, Desne Layer); (3) Discriminator. The following describes our motivation for adopting each unit.

Specifically, Time slice unit is placed at the input of the model to convert the MANETs topological data into a series of continuous static snapshots by a reasonable timestamp. It can compress data, reduce data redundancy, and improve the ability of information extraction. Based on the snapshots with the same interval, we can estimate the duration of each link in the future. In Generator, FastGCN extracts the spatial features of each static snapshot and feeds the results of network embedding into GRU to extract the network temporal features, to gain the continuous evolution law of MANETs. Dense Layer is used as a decoder to transform the extracted features back to the original space and generate prediction results. Considering the sparsity of the network, that is, the linked node pairs in the network are far smaller than the non-linked node pairs, which has a negative impact on the recovery of the topology, we use LSGANs to improve the generalization and generation ability of the model. In the process of model training, we use the observed network structures as the inputs of Discriminator to guide Generator to generate high-quality prediction results. In addition, the adoption of LSGANs is also due to the use of FastGCN. Based on node sampling, FastGCN greatly improves the training speed, but it will lead to the loss of information of some nodes. Therefore, we use LSGANs to improve the performance of the model. In the following, we describe each unit separately in detail.

### Time slice unit

The Time slice unit draws on the analysis method for time series data and divides the original continuously changing MANETs topological structure into a series of static snapshots through time slicing, which is used as the input data of the FastSTLSG for spatial and temporal feature extraction.

When the interval between consecutive slices is *t*, the number of network snapshots is $c = T/t$. At a slicing time $ti \in (t1,t2, \cdots ,tc)$, the elements $time_{uv}^{i}$ in the adjacency matrix ${\mathbf{A}}i$ of snapshot $Gi$ as link weights, represent the link duration of nodes *u* and *v* from the previous timestamp $ti$ to the next timestamp $ti + 1$.

We partition the MANETs topology into *c* discrete static weighted snapshots using the slicing time *t*. Obviously, the value of *t* directly affects the accuracy of the model. If *t* is too short, the input data is highly correlated, and the model tends to be insensitive to relatively independent new features, and prediction results are more biased to the data derived from these redundant features; if *t* is too long, input data contain too many new features, it is difficult for the model to extract the effective features from the large number of new features, resulting in low prediction accuracy.

In this paper, the dynamical behavior of the nodes in MANETs is regarded as a chaotic system^31^. The autocorrelation function method is used to determine a reasonable slice time length *t* by borrowing the coordinate delay phase space reconstruction technique in chaotic time series theory.

2 $$ R(t) = \frac{{\frac{1}{c}\sum\limits_{i = 1}^{c - 1} {\sum\limits_{u,v} {({\mathbf{A}}i + 1 - \overline{{{\mathbf{A}}i + 1}} )({\mathbf{A}}i - \overline{{{\mathbf{A}}i}} )} } }}{{\frac{1}{c}\sum\limits_{c = 1}^{c - 1} {\sqrt {[\sum\limits_{u,v} {({\mathbf{A}}i + 1 - \overline{{{\mathbf{A}}i + 1}} )^{2} ]} [\sum\limits_{u,v} {({\mathbf{A}}i - \overline{{{\mathbf{A}}i}} )^{2} } ]} } }} $$

where $R(t)$ is the correlation of each network snapshot when the slicing time is *t*, *c* represents the number of static snapshots, ${\mathbf{A}}i$ is the adjacency matrix of the *i*-th snapshot, $\overline{{{\mathbf{A}}i}}$ is the mean value of the elements in corresponding adjacency matrix ${\mathbf{A}}i$. For most self-learning models, the lower the correlation between the input data, the higher the independence of the data features. Related studies have shown that it is usually more appropriate to determine the value of $R(t)$ when it drops to $1/e$ for the first time in practical applications^22,32^, which is the basis for selecting the optimal slice time in this paper. After determining the appropriate *t*, the MANETs is transformed into *c* weighted network snapshots. We use a time window of length *s* to move smoothly over the snapshot sequence to obtain a series of consecutive snapshot sets of length *s*. So far, the subsequent training task of the FastSTLSG model is to learn a function that maps the input sequence to $Gt$ after given a sequence of snapshots $\{ Gt - s,Gt - s + 1, \cdots ,Gt - 2,Gt - 1\}$ of length *s*.

### FastGCN unit

In Section “GCN”, we have introduced the basic idea of GCN, which designs a subtle way to extract features from graph data and obtains embedding representations of the networks. Considering the drawbacks of poor scalability and high complexity of neighbor computation in GCN, we adopt FastGCN for spatial feature extraction of network snapshots. In FastGCN, the nodes in the snapshot are considered as independent identically distributed samples based on a probability distribution, and the convolution operation and loss function in GCN are transformed into an integral calculation of the embedding function based on a certain probability measure. The graph convolution operations and loss functions in the form of integrals can be approximated using Monte Carlo methods, and thus the nodes can be selected in batches for model training. Like inductive learning, the structure of the graph can be separated when the FastSTLSG is trained and predicted. The connection state of nodes can change, which effectively improves the generalization ability and scalability of the model in MANETs. In addition, compared with embedding methods such as GCN and GraphSAGE^33^, FastGCN can reduce the time complexity and improve the efficiency of the algorithm by using Monte Carlo method to approximate the computation of the convolution and loss function by node sampling. In FastGCN, the simplest way to sample nodes is to use a uniform distribution for sampling. It can also make selected nodes close to the real distribution through importance sampling, which can reduce the error caused by uniform sampling and improve the performance of the model. In summary, FastGCN can effectively solve the defects of ordinary GCN in spatial features extraction for large-scale and fast-changing MANETs and make the proposed model FastSTLSG more suitable for the practical application.

After slicing the network and obtaining a snapshot set $\left\{ {Gt - s,Gt - s + 1, \cdots ,Gt - 2,Gt - 1} \right\}$, *s* snapshots are fed into the FastGCN unit for spatial feature extraction, and the graph convolution operation of FastGCN is described below. If there exists a static snapshot *G* with weighted matrix **Z** in the snapshot sequence, *v* is a node in *G*. The convolution operation for *G* in FastGCN can be regarded as the integral calculation of the embedding function about node *v* and all other nodes in the upper layer, as shown in Eq. (3).

3 $$ \begin{gathered} {\tilde{\mathbf{h}}}^{{\left( {l + 1} \right)}} \left( v \right) \, = \int {{\hat{\mathbf{A}}}\left( {v, \, u} \right){\mathbf{h}}^{\left( l \right)} \left( u \right){\mathbf{W}}^{\left( l \right)} dP\left( u \right)} \hfill \\ {\mathbf{h}}^{{\left( {l + 1} \right)}} \left( v \right) \, = \, \sigma \left( {{\tilde{\mathbf{h}}}^{{\left( {l + 1} \right)}} \left( v \right)} \right),l = 0, \cdots , \, M - 1 \hfill \\ \end{gathered} $$

where *v* and *u* are nodes in snapshots, which are treated as independent random variables with the same probability measure; ${\hat{\mathbf{A}}}\left( {v, \, u} \right)$ is the element of the adjacency matrix ${\hat{\mathbf{A}}}$ at $\left( {v, \, u} \right)$; ${\mathbf{W}}^{(l)}$ is a set contained the parameters to be trained in *l-*th layer; ${\mathbf{h}}^{\left( l \right)} \left( u \right)$ is the embedding result of node *u* in *l-*th layer, which is calculated from the integral transformation of the embedding functions of all nodes in the upper layer. In particular,${\mathbf{h}}^{\left( 0 \right)} \left( v \right)$, the data of input layer, is the representation of the corresponding nodes on the characteristic matrix.

The convolution operation of graph is expressed in the form of integral function, which makes Eq. (3) to be approximately calculated by Monte Carlo method. In the *l*-th layer, the nodes are sampled independently and uniformly with probability *p* to obtain $tl$ sampling nodes $u_{1}^{(l)} , \cdots ,u_{{t_{l} }}^{(l)} \sim P$. Equation (3) can be expressed approximately as:

4 $$ \begin{gathered} {\tilde{\mathbf{h}}}_{{ t_{l + 1} }}^{(l + 1)} (v): = \frac{1}{{t_{l} }}\sum\limits_{j = 1}^{{t_{l} }} {{\hat{\mathbf{A}}}(v,u_{j}^{(l)} ){\mathbf{h}}_{{t_{l} }}^{(l)} (u_{j}^{(l)} ){\mathbf{W}}^{(l)} } \hfill \\ {\mathbf{h}}_{{t_{l + 1} }}^{(l + 1)} (v): = \sigma ({\tilde{\mathbf{h}}}_{{t_{l + 1} }}^{(l + 1)} (v)),l = 0, \cdots ,M - 1 \hfill \\ \end{gathered} $$

We uniformly sample nodes at each layer and finally get nodes $u_{t}^{(l)} ,i = 1, \cdots ,t_{l} ,l = 0, \ldots ,M - 1$. Uniformly sampling nodes in each *l*-row of ${\mathbf{H}}^{(l)}$ can be recursively represented as:

5 $$ \begin{gathered} {\mathbf{H}}^{(l + 1)} (v,:) = \sigma (\sum\limits_{j = 1}^{tl} {{\hat{\mathbf{A}}}} (u,u_{j}^{(l)} ){\mathbf{H}}^{(l)} (u_{j}^{(l)} ,:){\mathbf{W}}^{(l)} ) \hfill \\ l = 0, \ldots ,M - 1 \hfill \\ \end{gathered} $$

**Figure 3:**
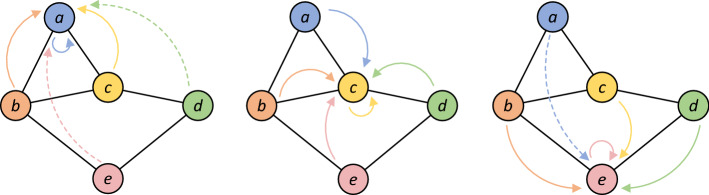
Convolution operation of node a, c, e in GCN.

**Figure 4:**
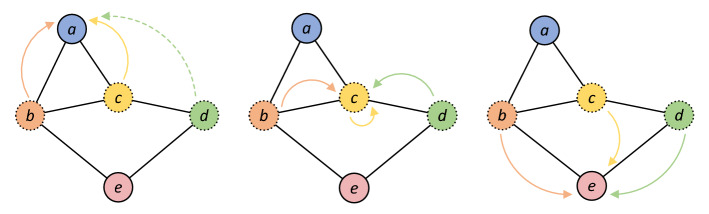
A batch convolution operation of node a, c, e in FastGCN with sampling node b, c, d.

Figures 3 and 4 show the comparison between GCN and FastGCN.

In GCN, the spatial feature of each node is obtained by the aggregation of feature from all nodes in the upper layer. The computational complexity of GCN is $o(n^{2} )$; In FastGCN, the large graph is divided into several small graphs through batch, and only $tl$ nodes are required to sample for convolution operation. The computational complexity of FastGCN is $o(n \times tl)$. Because of the sparsity of the network, $tl \ll n$, the training efficiency of FastGCN is greatly improved compared with GCN.

To further improve the ability of spatial feature extraction, we use importance sampling instead of uniform sampling in FastGCN, that is, each node is sampled by each probability distribution *Q*, which effectively reduces the sample variance and makes the distribution of sampled nodes closer to the real network structure. The Probability Mass Function (PMF) of each node in the network is shown in Eq. (6).

6 $$ q(u) = \left\| {{\hat{\mathbf{A}}}(:,u)} \right\|^{2} /\sum\limits_{u^{\prime} \in V} {\left\| {{\hat{\mathbf{A}}}(:,u^{\prime})} \right\|^{2} } ,u \in V $$

From Eq. (6), we can see that the PMF does not depend on parameter *l*, i.e., the sampling distribution is the same in all layers, and there is no need to update the sampling distribution function in real time as the training proceeds. Update Eqs. (5)–(7) after sampling $u1, \ldots ,ut$ nodes according to this distribution.

7 $$ {\mathbf{H}}^{(l + 1)} (v,:) = \sigma \left( {\sum\limits_{j = 1}^{{t_{l} }} {\frac{{{\hat{\mathbf{A}}}(v,u_{j}^{(l)} ){\mathbf{H}}^{(l)} (u_{j}^{(l)} ,:){\mathbf{W}}^{(l)} }}{{q(u_{j}^{(l)} )}}} ,u_{j}^{(l)} \sim q} \right),l = 0, \ldots ,M - 1 $$

In this paper, we use two layers for spatial feature extraction. The initial data ${\mathbf{H}}^{(0)}$ is the characteristic matrix **Z** that represents the link weights of the snapshot. In summary, FastGCN unit extracts spatial features based on adjacency matrixes $\{ {\mathbf{A}}t - s,{\mathbf{A}}t - s + 1, \ldots ,{\mathbf{A}}t - 2,{\mathbf{A}}t - 1\}$ of the input MANETs snapshots and the corresponding feature matrixes $\{ {\mathbf{Z}}t - s,{\mathbf{Z}}t - s + 1, \ldots ,{\mathbf{Z}}t - 2,{\mathbf{Z}}t - 1\}$, and then outputs a series of network embedding results $\{ {\mathbf{X}}t - s,{\mathbf{X}}t - s + 1, \ldots ,{\mathbf{X}}t - 2,{\mathbf{X}}t - 1\}$.

### GRU unit

After obtaining a series of the embedding results of snapshots series, capturing the long-term temporal correlation of each snapshot in time sequence is a key issue to predict the future structure of MANETs. RNNs can effectively process time series data, analyze the temporal characteristics of sequence data by using the temporal dependence of historical data, and complete the prediction of current and future moments. When the input data is a long sequence, the upper layers in RNN will be unable to learn the sequence features because of gradient disappearance. As a result, RNN only has the ability of short-term learning, it is difficult to use the previous historical information when handle the later data of the sequence. To solve the problem of short-term memory, Long Short-Term Memory (LSTM) network elaborately designs the gates to selectively change the flow of information in the historical sequence, decides whether the information in the historical sequence needs to be retained or discarded, which can keep the important features in front^34^. As a variant of LSTM, GRU has simpler structure and fewer training parameters, and can also avoid the gradient disappearance while retaining long-term sequence information^35^. In order to improve the training efficiency of the FastSTLSG and better apply it to the fast-changing MANETs, GRU is used in this paper to extract the temporal features of the network snapshot sequences.

In FastSTLSG, the embedding results after FastGCN unit $\{ {\mathbf{X}}t - s,{\mathbf{X}}t - s + 1, \ldots ,{\mathbf{X}}t - 2,{\mathbf{X}}t - 1\}$ are input to the GRU unit sequentially to capture the dynamic evolution of the MANETs in time sequence. The GRU can be described as a packaging module that repeatedly combines multiple multiplication gate cells (unit status, update gate, reset gate). Taking a time step *t* as an example, the inputs of the GRU unit are the input vector $xt$ at the current moment *t* and the state vector ${\mathbf{h}}t - 1$ at the previous moment $t - 1$. The statuses of gates in GRU are shown below.

8 $$ \begin{gathered} zt = \sigma ({\mathbf{W}}z \cdot [{\mathbf{h}}t - 1,{\mathbf{X}}t]) \hfill \\ rt = \sigma ({\mathbf{W}}r \cdot [{\mathbf{h}}t - 1,{\mathbf{X}}t]) \hfill \\ {\tilde{\mathbf{h}}}t = \tanh ({\mathbf{W}}h \cdot [rt * {\mathbf{h}}t - 1,{\mathbf{X}}t]) \hfill \\ {\mathbf{h}}t = (1 - zt) * {\mathbf{h}}t - 1 + zt * {\tilde{\mathbf{h}}}t \hfill \\ \end{gathered} $$

where, $zt$ and $rt$ represent the update gates and reset gates, respectively. $zt$ is used to control how much information from the previous state is brought into the current state. The larger the value of $zt$, the more information from the previous state is brought in. $rt$ is used to control how much information from the previous state is written to the current candidate set ${\tilde{\mathbf{h}}}t$. The smaller the value of $zt$, the less information from the previous state is added. ${\mathbf{W}}z$, ${\mathbf{W}}r$, ${\mathbf{W}}h$ are the parameters that GRU needs to train. $[ \cdot ]$ means two vectors are concatenated and $*$ means the product of matrices.

We choose GRU as the basic unit because it has fast convergence speed and can improve the training speed. A GRU needs to maintain three parameters, corresponding to update gates, reset gates and candidate sets respectively. In FastSTLSG, the output size and hidden size are equal. Therefore, the complexity of the GRU unit is $l \times 3 \times (ns \times ms + ns^{2} + ns)$, where $l$ is the number of GRU in the GRU unit, *ns* is the hidden size, *ms* is the input size.

To sum up, in FastSTLSG, the inputs of GRU unit are the embedding results of historical network snapshots $\{ {\mathbf{X}}t - s,{\mathbf{X}}t - s + 1, \ldots ,{\mathbf{X}}t - 2,{\mathbf{X}}t - 1\}$, and the output is the hidden layer of the last cell in GRU. Feed ${\mathbf{h}}t - 1$ to the fully connected layer, train and generate the predicted MANETs structure in the next time.

### LSGANs unit

In this paper, we employ LSGANs to further improve the ability of feature extraction and data generation. Generative Adversarial Networks (GAN), a generative model that has received much attention in recent years, have achieved widespread success in the fields of computer vision, image recognition, and natural language processing.

The core idea of GAN is derived from the Nash equilibrium from game theory, mainly composed of Generator (*G*) and Discriminator (*D*). The goal of *G* is to try to learn the real data distribution and generate fake data $G(z)$. The input of *D* is real data and fake data $G(z)$, and the output of *D* is a probability value that *D* identifies the input is from real data. The *D* wants to correctly distinguish whether the input data is from real data or from *G*. Meanwhile, the output of *D* will be fed back to *G* to guide *G*'s training. In the ideal case where the model reaches optimality, *D* is unable to distinguish the source of the input data. In the process of training, *G* and *D* will each update their own parameters to minimize the loss function. Through continuous iterative optimization, a Nash equilibrium state in finally reached, when the model is optimal. The objective function of GAN is defined as:

9 $$ \mathop {\min }\limits_{G} \mathop {\max }\limits_{D} ({\text{E}}x \sim pdata(x)[\log D(x)]) + {\text{E}}z \sim p(z)[\log (1 - D(G(z)))]) $$

where *x* is the input data and *z* represents the noise generated based on the probability distribution. However, the standard GAN has the problem of gradient disappearance. The training process is unstable, which leads to the unsatisfactory generation results. The reason for the problem is that although correct classification results can be obtained using cross-entropy, but some data that are classified as true and far away from the real samples will not be used to iterate anymore because they have successfully cheated *D*. It leads to saturation state easily because of gradient dispersion in *G*’s updating. LSGANs use least-squares loss to replace the cross-entropy loss in standard GAN, construct Pearson ${\chi }^{2}$ divergence instead of Jensen-Shannon (JS) divergence. It can finally construct a stable, efficient, and more powerful adversarial network with different distance metrics^36^. The specific loss function and training procedure of LSGANs are described in Section “Loss function”.

In FastSTLSG, we consider the adversarial between generator and discriminator as a minimax game in LSGANs. The input of *G* is a sequence of historical network snapshots, and the output is the predicted future network structure. *D* uses the real future network structure as a condition to discriminate whether the generated prediction results come from *G* or not, until the training is stable. When the prediction results generated by *G* can deceive *D*, it is considered that *G* is high quality to complete the link prediction for MANETs.

#### Generator

As shown in Fig. 2, the generator in FastSTLSG is composed of FastGCN, GRU and Dense Layer unit. The FastGCN unit extracts the spatial feature of the historical network snapshots, and its inputs are a adjacency matrices $\{ {\mathbf{A}}t - s,{\mathbf{A}}t - s + 1, \ldots ,{\mathbf{A}}t - 2,{\mathbf{A}}t - 1\}$ and feature matrices $\{ {\mathbf{Z}}t - s,{\mathbf{Z}}t - s + 1, \ldots ,{\mathbf{Z}}t - 2,{\mathbf{Z}}t - 1\}$, its outputs are the embedding results $\{ {\mathbf{X}}t - s,{\mathbf{X}}t - s + 1, \ldots ,{\mathbf{X}}t - 2,{\mathbf{X}}t - 1\}$. The embedding results are transformed into vectors and then input into GRU unit. The GRU unit is used to extract the temporal feature of the historical snapshots by using the powerful sequential data extraction ability. The outputs of GRU unit are the state vectors of hidden layer $\{ {\mathbf{h}}t - s,{\mathbf{h}}t - s + 1, \ldots ,{\mathbf{h}}t - 2,{\mathbf{h}}t - 1\}$. The vector in the last time stage ${\mathbf{h}}t - 1$ is input to Dense Layer. The output of Dense Layer is ${\tilde{\mathbf{A}}}t$, the prediction result for MANETs in time *t*. To sum up, the input and output of *G* can be simply expressed as:

10 $$ {\tilde{\mathbf{A}}}_{t} = G({\mathbf{A}}_{t - 1}^{t - s} ,{\mathbf{Z}}) $$

where ${\mathbf{A}}_{t - 1}^{t - s} = \{ {\mathbf{A}}t - s,{\mathbf{A}}t - s + 1, \ldots ,{\mathbf{A}}t - 2,{\mathbf{A}}t - 1\}$ is the historical MANETs structure from $t - s$ to $t - 1$;${\tilde{\mathbf{A}}}t$ is the predicted MANETs structure at moment *t* by FastSTLSG.

#### Discriminator

The discriminator *D* is used to discriminate whether the input prediction network is generated by *G*. *D* consists of a Dense Layer and an activation function. During model training, the output of *G* ${\tilde{\mathbf{A}}}t$ and the real network adjacency matrix ${\mathbf{A}}t$ are alternately fed into *D*. $\{ {\tilde{\mathbf{A}}}t,{\mathbf{A}}t\}$ are used as the inputs of the Dense Layer for training, and the output is calculated through the activation function to complete the discrimination. It is worth noting that the input of Dense Layer is in the form of vector, but $\{ {\tilde{\mathbf{A}}}t,{\mathbf{A}}t\}$ are $V \times V$ dimensional matrixes. It needs to transform $\{ {\tilde{\mathbf{A}}}t,{\mathbf{A}}t\}$ into vectors and fed them into Dense Layer. In summary, the input and output of *D* can be simply expressed as:

11 $$ D({\mathbf{A}}) = \sigma (({\mathbf{AW}}1 + b1){\mathbf{W}}2 + b2) $$

where $\{ {\tilde{\mathbf{A}}}t,{\mathbf{A}}t\}$, $\{ {\mathbf{W}}1,b1\}$, $\{ {\mathbf{W}}2,b2\}$ are the weight parameters and bias parameters to be trained in the dense layer and the output layer respectively.

The computational complexity of LSGANs unit is introduced below. The computational complexity of LSGANs is related to the network size, which is $o(nA^{2} )$, where $nA$ is the number of all elements in matrix ${\mathbf{A}}t$.

### Loss function

In the process of training the *G* and *D*, one unit is fixed, and the other unit's parameters are updated by alternating iterations. The loss function of FastSTLSG training is divided into adversarial loss and reconstruction loss, which are described below.

#### Adversarial loss

The adversarial loss is the loss function of *G* and *D* in the adversarial process. LSGANs use least squares loss function to penalize samples which are discriminated to be true and far away from the decision boundary. It can drag the false samples far away from the decision boundary into the decision boundary, to improve the quality of the *G*. The adversarial loss function is expressed as follows:

12 $$ \begin{gathered} L(G) = \frac{1}{2}{\text{E}}{\mathbf{A}}_{t - 1}^{t - s} \sim pdata({\mathbf{A}}),Z \sim p({\mathbf{Z}})[(D(G({\mathbf{A}}_{t - 1}^{t - s} ,{\mathbf{Z}})) - d)^{2} ] \hfill \\ L(D) = \frac{1}{2}{\text{E}}{\mathbf{A}}t \sim pdata({\mathbf{A}})[(D({\mathbf{A}}t) - b)^{2} ] + \frac{1}{2}{\text{E}}{\mathbf{A}}_{t - 1}^{t - s} \sim pdata({\mathbf{A}}),{\mathbf{Z}} \sim p({\mathbf{Z}})[(D(G({\mathbf{A}}_{t - 1}^{t - s} ,{\mathbf{Z}})) - a^{2} ] \hfill \\ \end{gathered} $$

where $pdata({\mathbf{A}})$ is the distribution of snapshots; ${\mathbf{A}}_{t - 1}^{t - s}$ represents the snapshots from $t - s$ to $t - 1$; ${\mathbf{A}}t$ represents the snapshots at *t*; ${\tilde{\mathbf{A}}}t = G({\mathbf{A}}_{t - 1}^{t - s} ,{\mathbf{Z}})$ represents the prediction result of the MANETs; the constants *a* and *b* are the encoding of the real network data and the topology generated by the *G*, respectively; *c* is the encoding set by the *D* to treat the network structure generated by the *G* as the real network. When $b - d = 1$ and $b - a = 2$, the objective function is equivalent to Pearson ${\chi }^{2}$ divergence. In FastSTLSG, $a = - 1$, $b = 1$, $d = 0$. Finally, the adversarial loss of the FastSTLSG is as follows:

13 $$ \begin{gathered} L(G) = \frac{1}{2}{\text{E}}{\mathbf{A}}_{t - 1}^{t - s} \sim pdata({\mathbf{A}}),{\mathbf{Z}} \sim p({\mathbf{Z}})[(D(G({\mathbf{A}}_{t - 1}^{t - s} ,{\mathbf{Z}})))^{2} ] \hfill \\ L(D) = \frac{1}{2}{\text{E}}{\mathbf{A}}t \sim pdata({\mathbf{A}})[(D({\mathbf{A}}t) - 1)^{2} ] + \frac{1}{2}{\text{E}}{\mathbf{A}}_{t - 1}^{t - s} \sim pdata({\mathbf{A)}},{\mathbf{Z}} \sim p({\mathbf{Z}})[(D(G({\mathbf{A}}_{t - 1}^{t - s} ,{\mathbf{Z)}}) + 1)^{2} ] \hfill \\ \end{gathered} $$

In FastSTLSG, *G* wants the prediction result to be as close to the real result as possible, and *D* wants the discriminative power to be stronger, so the adversarial loss function $L(G)$, $L(D)$ needs to be minimized.

#### Reconstruction loss

We need the prediction result ${\tilde{\mathbf{A}}}t$ to be as close as possible to the actual network ${\mathbf{A}}t$. To improve the accuracy of the prediction, we use Mean Squared Error (MSE) to measure the similarity between ${\tilde{\mathbf{A}}}t$ and ${\mathbf{A}}t$. The reconstruction loss is as follows.

14 $$ L{\text{recons}} = \left\| {({\mathbf{A}}t - {\tilde{\mathbf{A}}}t)} \right\|_{F}^{2} $$

However, due to the sparsity of the network, that is, the zero elements in the adjacency matrix of the network are much larger than the non-zero elements, which will lead to *G* more inclined to generate many zero elements, making the loss function unable to converge or even over fit. To solve the problem of sparsity in the network, we use penalty matrix **P** to impose greater penalty on the non-zero elements in ${\mathbf{A}}t$. The improved reconstruction loss is as follows:

15 $$ L{\text{recons}} = \left\| {({\mathbf{A}}t - {\tilde{\mathbf{A}}}t) \odot {\mathbf{P}}} \right\|_{F}^{2} $$

where $\odot$ is Hadamard Product. If the element in ${\mathbf{A}}t$ satisfies ${\mathbf{A}}t(u,v) = 0$, then ${\mathbf{P}}(u,v) = 1$, otherwise ${\mathbf{P}}(u,v) = \beta > 1$. By setting **P** to impose more penalties on the non-zero elements in ${\mathbf{A}}t$, and ${\mathbf{A}}t$ is guided to not generate zero elements in the corresponding positions in ${\tilde{\mathbf{A}}}t$ as much as possible. We also further prevent overfitting by introducing L2 regularization, which punishes the squared terms of all parameters and imposes a greater penalty on large weights.

16 $$ L{\text{reg}} = \frac{\lambda }{2}\left\| {{\mathbf{W}}G} \right\|_{2}^{2} $$

where ${\mathbf{W}}G$ represents the matrix containing all parameters to be trained in *G*; $\lambda$ is the coefficient that controls the penalty effect of the L2 regular term. In summary, combined with Eqs. (13), (15), and (16), the overall loss function of FastSTLSG is shown in Eq. (17).

17 $$ L{\text{total}} = \mathop {\min }\limits_{G} \mathop {\min }\limits_{D} L(G,D) + \alpha L{\text{recons}} + L{\text{reg}} $$

In the model training, the Adam optimizer is used to alternately update the parameters matrix of *G* and *D*, that is, ${\mathbf{W}}G$ and ${\mathbf{W}}D$. The iterations are terminated after *G* and *D* reach equilibrium. After the training is completed, the historical MANETs topology can be input into *G* to obtain the prediction of the MANETs at future moments, thus achieving link prediction for MANETs. The pesudocode of the FastSTLSG proposed in this paper is shown in Table 2.

**Table 2:**
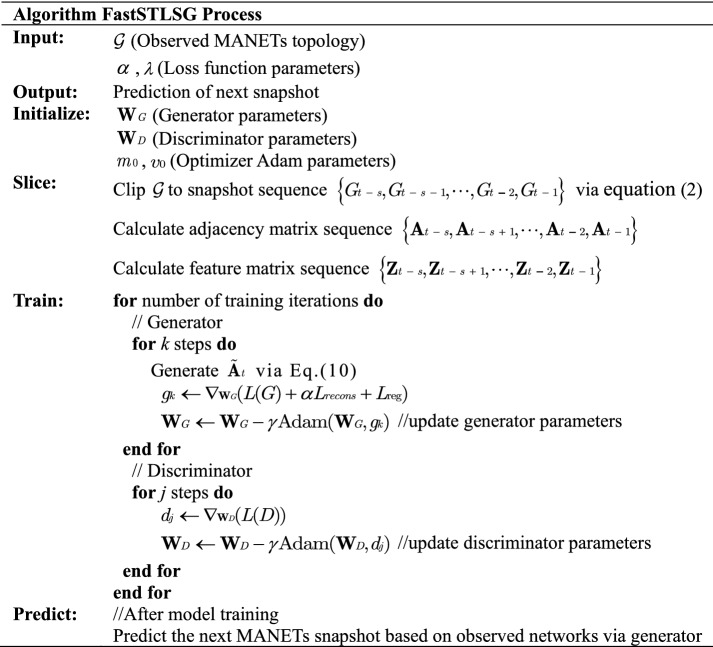
Algorithm FastSTLSG process.

In addition, we declare that in this paper, all methods are carried out in accordance with relevant guidelines and regulations.

## Experiment

The proposed FastSTLSG then is evaluated on four bench-mark datasets, compared with eight baseline methods.

### Datasets

1. CONTACT (http://konect.cc/files/download.tsv.contact.tar.bz2): This dataset records an undirected human contact network. The trace records all contact information and data transfer between a group of users who carry wireless devices at various locations. In the dataset, a node represents a user; an edge between two users means that there is a contact between two proximate devices.
2. HYCCUPS (https://crawdad.org/upb/hyccups/20161017): This dataset is collected from trace of wireless contacts and users’ connections at the Politehnica University of Bucharest in the period from March to May 2012. 72 participants used an Android application named HYCCUPS Tracer to collect mobile interaction information in the background. It records sensor data and connectivity information between smart devices and other wireless access devices based on AllJoyn framework.
3. ASTURIESER (https://crawdad.org//download/oviedo/asturies-er/asturies-er-1year-mobility.csv.gz): The dataset comprises mobility traces and connectivity information of around 229 vehicles and helicopters in the regional Fire Department of Asturias, Spain, collected over one year. The datacenter collects devices current positions, status, contacts, etc., every 30 s. The threshold of communication distance between different devices is 10 m, 50 m or 200 m. Proximate nodes can exchange the information when the distance between them is below the threshold.
4. ROTAXI (https://crawdad.org//download/roma/taxi/taxi_february.tar.gz): This dataset is derived from the real motion traces from approximately 320 taxis with GPS devices in Roma, Italy, for the duration of one month. Each taxi driver uses the location manager software based on GPS to retrieve longitude and latitude coordinates of current location periodically. The dataset also records time duration, speed, status, and contact information among devices.

The experiment is carried out with the above networks. The basic details of four datasets are shown in Table 3.

**Table 3:** Basic details of four datasets.

Dataset	Device	Size	Duration (days)	Mode	Sampling frequency
CONTACT	iMote	274	4.0	Bluetooth	/10 s
HYCCUPS	Android phone	72	63.0	Wi-Fi	/80 s
ASTURIESER	Vehicle, Helicopter	229	365.0	GPS	/30 s
ROTAXI	Vehicle	320	30.0	GPS	/7 s

### Baseline methods

To evaluate the performance of the proposed FastSTLSG, we carry out extensive experiments and compare it with several widely used baseline methods. In particular, the baselines are shown as follows.

1. Common Neighbors (CN)^37^: CN is one of the most widely used metrics to evaluate the performance of link prediction. The probability of two nodes to generate links in the future is positively correlated with the number of common neighbors they had in the past.
2. Deep Dynamic Network Embedding (DDNE)^17^: Like autoencoder, DDNE uses an RNN and interaction proximity. It can capture the nonlinear transformation characteristics of networks and analyze the interaction information of nodes in an evolution period.
3. Node2Vec^38^: As an embedding method, Node2Vec uses random walk sampling to get the combination of nodes, and then mines the connection relationship of nodes to obtain the low dimensional vector representation. The existence probability of links is related to the similarity of vectors.
4. Temporal Matrix Factorization (TMF)^39^: TMF can explicitly transform the network into a function with time parameters based on matrix factorization. It has great advantages in dealing with dynamic network timing tasks, such as predicting the evolution of networks with time series.
5. E-LSTM-D^6^: E-LSTM-D is a novel deep learning model for dynamic network, which is composed of an encoder, LSTMs, and a decoder. It can learn both structure and time characteristics of networks with different scales in an end-to-end framework.
6. GTRBM^40^: As a supervised method, GTRBM combines the idea of gradient enhanced decision tree (GBDT) and Time Restricted Boltzmann machine (TRBM) and captures the topological characteristics of networks. It has enough hidden layers which can model the dynamic nonlinear transformation.
7. DGFT^20^: DGFT is an advanced deep generative framework for temporal link prediction in dynamic networks.
8. GGAN^21^: GGAN is a link prediction model which can extract features from weighted dynamic network. Its advantage is that it can mine nonlinear temporal data.

The basic parameters of the eight baseline methods are shown in Table 4.

**Table 4:** Parameters of the eight baseline methods.

Baseline methods	Basic parameters
CN	–
DDNE	Learning rate: $1e - 2$, Weight decay: $5e - 4$
Node2Vec	Optimal transfer probability *p* and *q* are obtained by grid search in $(0.25,0.5,1,1.5,2)$
TMF	Order of time-dependent matrix:2, Decay function parameter: 0.3, latent dimensional parameter: 10
E-LSTM-D	Learning rate: $1e - 3$, Weight decay: $1e - 4$
GTRBM	Learning rate: $1e - 2$, Weight decay: $5e - 4$
DGFT	Dropout rate: 0.2, Order of time-dependent matrix: 2
GGAN	Hidden space size: 32, Learning rate: $1e - 2$

### Quantitative evaluation metrics

1. Area Under the Curve (AUC)^41^: AUC is the area under the Receiver Operating Characteristic (ROC) curve, which is used to measure the accuracy of link prediction method generally. After model training, AUC can get the score of the existence probability of each link in the network by calculating and comparing the score of the link in the test set and the score of the non-existent link.
  18 $$ {\text{AUC}} = \frac{n^{\prime} + 0.5n^{\prime\prime}}{n} $$
  In *n* independent comparison, the number of times that the score of higher weight link is greater than that of lower weight link is $n^{\prime}$, and the number of same scores is $n^{\prime\prime}$.
2. Geometric Mean AUC (GMAUC)^42^: As a unified evaluation metric, GMAUC is composed of the average value of AUC and Precision Recall AUC (PRAUC). AUC focuses on analyzing previous links, GMAUC is used to solve class imbalance problem caused by new links. The formula of GMAUC is as follows.
  19 $$ {\text{GMAUC = }}\sqrt {\frac{{{\text{PRAUCnew}} - \frac{P}{P + N}}}{{1 - \frac{P}{P + N}}} \cdot 2({\text{AUC}} {\text{prev}} - 0.5)} $$
  where *P* and *N* represent the number of correct and incorrect predictions for the newly generated links.
3. Root Mean Squared Error (RMSE): RMSE is the arithmetic square root of the mean square of the difference between the estimated value and the real value, which can evaluate the change of the data. The smaller value of RMSE signifies the more accurate prediction model.
  20 $$ {\text{RMSE = }}\frac{1}{T}\sum\limits_{t = 1}^{T} {\sqrt {\frac{1}{M}\left\| {{\mathbf{A}}_{t} - \widetilde{{{\mathbf{A}}_{t}}}} \right\|F} } $$
  where *M* represents the number of elements in the matrix ${\mathbf{A}}_{t}$, *T* represents the number of predictions.
4. Non-existent Rate (NER): RMSE is not sensitive to the existence of links. For example, the difference between 0 and 1 should be more significant than the difference between 1 and 2, although the RMSE is same in both cases. NER is more intolerable to mistakenly estimate the nonexistent link as existing.

21 $$ {\text{NER = }}\frac{1}{T}\sum\limits_{t = 1}^{T} {\sqrt {\frac{1}{M}{\text{num}}(\widetilde{a_{t}} \ne 0\,{\text{if}}\,at = 0\,{\text{or}}\,\widetilde{a_{t}} = 0\,{\text{if}}\,at \ne 0)} } $$

where $a_{t}$ and $\widetilde{a_{t}}$ are elements of ${\mathbf{A}}_{t}$ and $\widetilde{{{\mathbf{A}}_{t}}}$ respectively. In practice, the threshold is 0.01.

### Experimental details

#### Experimental environment

We use Pytorch to build the model based on Python. In addition, we use CUDA platform to implement GPU parallel computing and cuDNN to improve the training speed of deep neural network.

#### Time slice unit

In Section “Time slice unit”, we have introduced how to slice the MANETs into a series of interval snapshots as the inputs of generator in LSGANs, and the link weights of the snapshots represent the time of device contact. The time interval between two snapshots is determined by the corresponding *t* when the $R(t)$ in Eq. (2) first drops to $1/e$.

Before feeding data to the generator, every ten consecutive snapshots constitute a time window, that is $s = 10$. To be more precise, $\{ Gt - 10,Gt - 9, \cdots ,Gt - 2,Gt - 1\}$ is the input and $Gt$ is the output to be predicted. Then, we smoothly slide the time window for rest snapshots and divide all the samples according to a certain proportion, 80% as the training set, and the rest as the testing set.

#### FastGCN unit

In each dataset, the nodes are divided into several batches. FastGCN contains three layers, the first layer samples 30% nodes, the second layer samples 30% nodes, and the third layer does not sample. The initialization parameters comply with Glorot uniform distribution, the number of units in hidden layer is set to 16 for the CONTACT and 32 for the other datasets, maximum Chebyshev polynomial degree is set to 3, the Dropout is set to 0, the number of output features is set to 64. Significantly, the input and output of FastGCN are matrices, which need to be converted into vectors before being input into GRU.

#### GRU unit

The initialization parameters of GRU comply with orthogonal distribution, the Dropout is set to 0.2, the bias state of the hidden layer is true, the sequence length is set to 10, the hidden size is set to 16 for the CONTACT and 32 for the other datasets.

#### LSGANs unit

$\lambda$ as the coefficient of L2 regularization is set to $1e - 4$, $\alpha$ as the coefficient of reconstruction loss is set to 0.3. The parameters of generator and discriminator are updated alternately by Adam optimizer and the learning rate for training of generator and discriminator is set to $1e - 3$. In the process of training, epoch is set to 200. The training will be terminated in advance if the value of loss function does not decrease after 10 consecutive epochs.

### Result analysis

The performance of the FastSTLSG proposed in this paper with eight benchmark methods are shown in Tables 5, 6, 7 and 8. The AUC, GMAUC, RMSE and NER are used to reveal the ability of each model to capture the spatial–temporal evolution characteristics and predict the future links for MANETs.

**Table 5:** Performances on AUC for different methods.

Method	CONTACT	HYCCUPS	ASTURIESER	ROTAXI
CN	0.6574	0.7032	0.5984	0.6926
DDNE	0.8485	0.8295	0.7896	0.8163
Node2Vec	0.7938	0.8512	0.6412	0.6123
TMF	0.8924	0.8637	0.8491	0.8764
E-LSTM-D	0.9136	0.8965	0.8026	0.9034
GTRBM	0.8126	0.8268	0.8772	0.7415
DGFT	0.9234	0.8792	0.8642	0.9421
GGAN	0.9167	0.8942	0.8902	0.9003
FastSTLSG	**0.9427**	**0.9136**	**0.9231**	**0.9542**

Significant values are in bold.

**Table 6:** Performances on GMAUC for different methods.

Method	CONTACT	HYCCUPS	ASTURIESER	ROTAXI
CN	0.6631	0.6412	0.5136	0.6291
DDNE	0.8075	0.8354	0.8498	0.8367
Node2Vec	0.7168	0.7064	0.6584	0.5697
TMF	0.9015	0.8643	0.8231	0.8841
E-LSTM-D	0.9374	0.9016	0.8262	0.9231
GTRBM	0.8378	0.8911	0.8165	0.7566
DGFT	0.9612	0.9014	0.8953	0.9026
GGAN	0.9125	0.8879	0.9127	0.8973
FastSTLSG	**0.9671**	**0.9197**	**0.9456**	**0.9534**

Significant values are in bold.

**Table 7:** Performances on RMSE for different methods.

Method	CONTACT	HYCCUPS	ASTURIESER	ROTAXI
CN	0.3892	10.051	0.6764	4.6421
DDNE	0.2432	6.2980	0.4112	1.4312
Node2Vec	0.2145	7.1359	0.5058	2.699
TMF	0.1745	2.1864	0.3573	1.9845
E-LSTM-D	0.1871	4.3198	0.3196	1.3523
GTRBM	0.2035	8.3554	0.5418	2.6942
DGFT	0.1568	2.0351	0.4563	0.9872
GGAN	0.1892	1.6895	0.3251	1.1263
FastSTLSG	**0.1258**	**1.5642**	**0.1894**	**0.4581**

Significant values are in bold.

**Table 8:** Performances on NER for different methods.

Method	CONTCT	HYCCUPS	ASTURIESER	ROTAXI
CN	0.3231	0.5136	0.2664	0.2755
DDNE	0.0612	0.1264	0.0676	0.0942
Node2Vec	0.2694	0.2468	0.3158	0.2453
TMF	0.1236	0.0979	0.2697	0.0639
E-LSTM-D	0.0972	0.1023	0.0482	0.0435
GTRBM	0.1298	0.1920	0.0841	0.0634
DGFT	0.0786	0.0812	0.0752	0.0545
GGAN	0.0964	0.0546	0.0325	0.0768
FastSTLSG	**0.0213**	**0.0347**	**0.0124**	**0.0278**

Significant values are in bold.

From the Tables 5, 6, 7 and 8, FastSTLSG achieves the optimal prediction performance in each network compared with the baseline models, which proves the effectiveness of the model proposed in this paper. If the baseline metrics are AUC and GMAUC, CN and Node2Vec perform poorly compared to other models, and the scores of CN and Node2Vec in the four networks are less than 0.8 in most cases (the scores of other models are greater than 0.8 in most cases). We believe that the low prediction accuracy of CN and Node2Vec is due to the following two reasons: (1) The central idea of CN and Node2Vec is to capture the spatial structure characteristics of static networks, ignoring the temporal evolution pattern of dynamic networks, and the models proposed for static networks are not applicable to dynamic network link prediction such as MANETs; (2) CN focuses on mining the neighbor information of nodes at both ends of the links, and Node2Vec uses hyperparameters to gain different wandering paths. Both models lack the ability to mine the deep structure features and global features of MANETs. Other benchmark models have better performance compared to CN and Node2Vec because they consider the evolution characteristics of MANETs, and their AUC and GMAUC scores do not differ much. The AUC and GMAUC scores of FastSTLSG are higher than those of benchmark prediction models, which means that FastSTLSG outperforms existing benchmark models in terms of overall qualitative prediction performance.

Further, the RMSE quantitatively measures the prediction performance using the mean squared error between the predicted results and the true results, and NER is used to measure probability of error link prediction. The RMSE and NER metrics focus on revealing the generative ability of the model, i.e., the accuracy of the prediction results. We find that the RMSE and NER of CN and Node2Vec are still poor, which means that the error is high. We consider that this is because CN lacks the ability to characterize nonlinearities, and nonlinear regression of Node2Vec is not applicable to feature extraction of dynamic networks. In this paper, we propose the FastSTLSG, which draws on the idea of minimax adversarial training, and uses LSGANs to improve the quality of the generator to predict the topology at the next moment. Compared with the benchmark models, the FastSTLSG obtains the prediction results closer to the real network structure.

**Figure 5:**
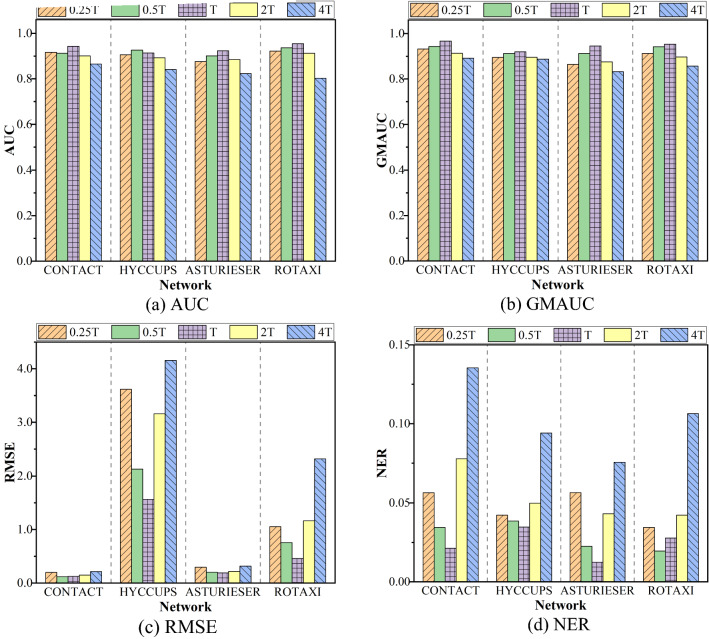
Influence of different slice durations on prediction accuracy.

Figure 5 shows the impact of different slice durations on the prediction accuracy of FastSTLSG. Based on calculating the appropriate slicing duration *T* using Eq. (2), we respectively calculate the value of 0.25, 0.5, 2, and 4 multiples of *T*, and repeat the experimental steps to calculate the prediction performance of FastSTLSG at different slicing durations. The experimental results are shown in Fig. 5. It can be seen from the figure that, in most cases, compared to other slicing durations, network snapshots based on *T* time slices can obtain the optimal performance. When the evaluation criteria are AUC and GMAUC, different slice durations have little effect on accuracy of FastSTLSG, the scores corresponding to 0.25* T*, 0.5* T*, 2* T*, and 4* T* are all slightly lower than *T*, but higher than 0.8. When the evaluation criteria are RMSE and NER, the slice durations have a greater effect on the prediction accuracy in most networks, and the shorter or longer the time window, the better the prediction is not necessarily better. This is consistent with our expectation. If the interval of snapshots is too short, the model is insensitive to relatively independent new features, and a shorter interval means less information about the spatial and temporal features, which causes difficulties in predicting network evolution; If the interval is too long, too many new spatial–temporal features overlap in the same network snapshot, which causes great difficulties for the model to extract effective features and leads to lower prediction accuracy. From the results, the FastSTLSG uses the autocorrelation method in phase space reconstruction of chaotic systems to determine a reasonable slice duration. Compared with other baseline durations, it can obtain the optimal or sub-optimal performance, which verifies the reasonableness of the slice duration selected by FastSTLSG.

To analyze the efficiency of the proposed model FastSTLSG and other deep learning frameworks, we record the training time of multiple deep learning models running on the experimental networks. The training time in one epoch is shown in Table 9. From Table 9, compared with other models, our model FastSTLSG costs the least training time in the four networks. This shows that our model is the most efficient in processing dynamic graph data and can better adapt to the MANETs with rapid change of topology.

**Table 9:** Training time in one epoch in four deep learning models.

Method	CONTACT	HYCCUPS	ASTURIESER	ROTAXI
E-LSTM-D	10 m 13 s	5 m 28 s	20 m 47 s	23 m 12 s
DGFT	4 m 17 s	2 m 10 s	9 m 13 s	7 m 19 s
GGAN	5 m 9 s	1 m 52 s	7 m 46 s	8 m 1 s
FastSTLSG	**58 s**	**23 s**	**2 m 49 s**	**2 m 3 s**

Significant values are in bold.

**Figure 6:**
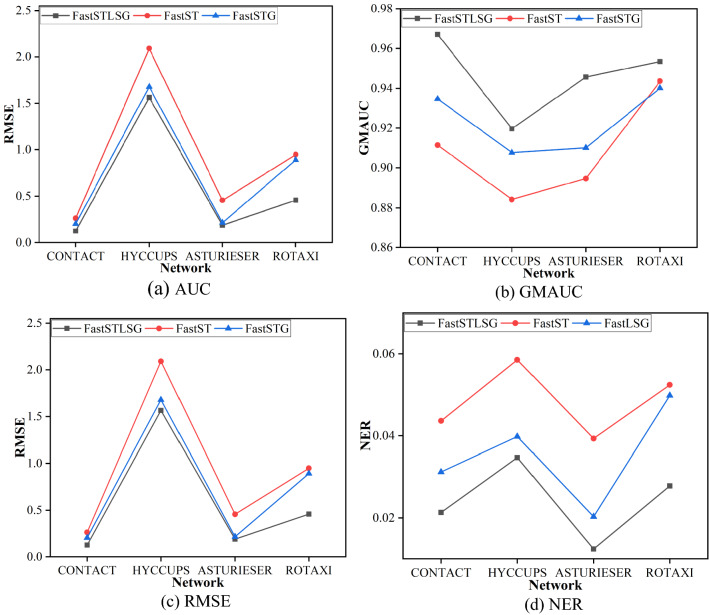
Performances on different MANETs for FastSTLSG, FastST and FastG.

In addition, to further verify the optimizing effect of LSGANs unit on the generator in the FastSTLSG model, we remove the discriminator from the FastSTLSG, and delete the adversarial loss in the loss function, and keep $L{\text{recons}}$ and $L{\text{reg}}$. The FastSTLSG with stripped LSGANs is renamed as FastST. To further illustrate the advantages of LSGANs, we replace LSGANs unit with standard GAN, modify the adversarial loss to Eq. (9), and keep $L{\text{recons}}$ and $L{\text{reg}}$ in loss function. The new model with standard GAN is renamed as FastSTG. The experimental steps are repeated to analyze the prediction performance of FastST and FastSTG for MANETs. The link prediction performance results of FastSTLSG, FastST, FastSTG models are shown in Fig. 6.

From experimental results, the performance of FastSTLSG decreases in all metrics after removing the LSGANs unit or replacing LSGANs unit with standard GAN. Compared with FastST, the AUC and GMAUC of our model increase 3.8% and 5.2%, respectively, the RMSE and NER of our model decrease 46.8% and 51.3%, respectively. Compared with FastSTG, the AUC and GMAUC of our model increase 2.1% and 2.5%, respectively, the RMSE and NER of our model decrease 26.4% and 30.3%, respectively. This shows that LSGANs unit overcomes the defects of standard GAN, extracts dynamic graph features more effectively than GAN, and obtains better prediction accuracy. LSGANs can facilitate FastSTLSG to generate high-quality network topology results through effective learning for spatial features. Thus, we further demonstrate the contribution of LSGANs unit to FastSTLSG.

Based on the above experimental results, the proposed model FastSTLSG can effectively extract the spatio-temporal features in MANETs based on obtaining the reasonable slice time. LSGANs unit is used to improve the generation ability of the prediction model. Compared with the existing methods, FastSTLSG has better accuracy, which shows the effectiveness and excellent performance.

### Case study

Our proposed FastSTLSG model can effectively extract the spatio-temporal features in the temporal networks and can achieve the link prediction task of MANETs efficiently and with high quality. Extensive experiments on several datasets demonstrate the superior performance of our model compared to the baseline methods. In fact, FastLSG can be applied not only in MANETs, but also in any topology graph with temporal structure to accomplish link prediction. In the following, we take the traffic flow prediction task as a case study to briefly demonstrate the high-quality prediction results of our model.

Traffic flow prediction aims to predict future traffic based on previous traffic flows. Multiple roads and multiple cameras in a region record rich spatial information, and the traffic flows recorded by intervals have temporal features^43^. Therefore, we can consider the traffic flow as a spatio-temporal topology graph and use the temporal and spatial information in the data to predict the future traffic flow of different roads in the region.

**Figure 7:**
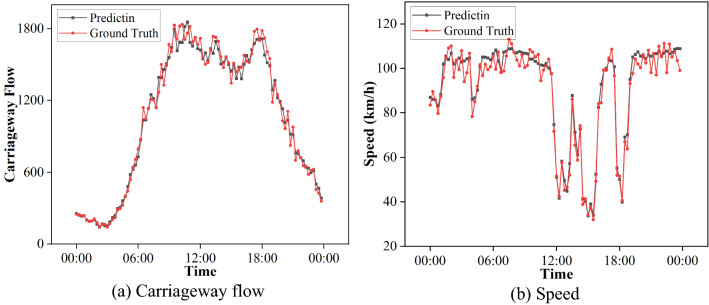
Link prediction for carriageway flow and speed.

MIDAS (http://tris.highwaysengland.co.uk/detail/trafficflowdata) is a Traffic Flow Data, which records the traffic flow on the UK highways every fifteen minutes. We extract 64 detectors as nodes to form a traffic network to predict the traffic flow information. The prediction result of visualizing one of the roads is shown in Fig. 7.

It can be seen that FastLSG can accurately predict the number of vehicles and their speed in the roads. The accurate prediction results can provide a reference for traffic management to establish traffic planning and vehicle owners to plan their trips. FastLSG can effectively capture the spatial topology and temporal dependence of the graph. This case shows that our model can handle not only MANETs, but also diverse temporal networks. It demonstrates the excellent prediction performance of our model.

## Conclusion

In this paper, we propose a link prediction model named FastSTLSG to solve the problem of graph prediction with spatial and temporal features and apply it to MANETs. Specifically, to predict the evolution pattern of the MANETs, the FastSTLSG successfully extracts the spatial–temporal properties of the topology by using the historical network information. The model learns not only low-dimensional embedding and nonlinear structure, but also temporal correlations between continuous network snapshots. The main contributions of this paper include: (1) An efficient spatio-temporal feature extraction model framework for MANETs is proposed; (2) Using chaotic time theory to calculate the network slice time, and the link duration is taken as the prediction target; (3) LSGANs unit is used to improve the ability of the model to recover the network structure from the low dimensional dense embedding vectors, and further improve the prediction accuracy of the model.

The FastSTLSG model is applicable not only to MANETs, but also to all graph data with spatio-temporal features, especially large-scale networks. In the next step, we will study about the heterogeneous dynamic networks, and further expand the application of the model proposed in this paper.

## Data Availability

The data can be download in CONTACT (http://konect.cc/files/download.tsv.contact.tar.bz2), HYCCUPS (https://crawdad.org/upb/hyccups/20161017), ASTURIESER (https://crawdad.org//download/oviedo/asturies-er/asturies-er-1year-mobility.csv.gz), ROTAXI (https://crawdad.org//download/roma/taxi/taxi_february.tar.gz).
